# ‘It Is of Course About Being Human Together’: Co‐Development of a Primary Progressive Aphasia Awareness Campaign to Enhance Understanding of Speech and Language Therapy

**DOI:** 10.1111/1460-6984.70324

**Published:** 2026-09-08

**Authors:** Anna Volkmer, Rosemary Townsend, Bethany Anthony, Sharon M. Antonucci, Petronilla Battista, Sandrine Basaglia‐Pappas, Jeanne Gallée, Stephanie Grasso, Maya L. Henry, Peter Just, Nomiki Karpathiou, Becky Khayum, Monica Lavoie, Helen Moffat, Michael Petley, Philip Robinson, Kiiya Shibata, Cathleen Taylor‐Rubin, Vanessa Ward, Jason D. Warren, Nikki Zimmerman, the BCPPA Patient and Public Involvement Group

**Affiliations:** ^1^ Division of Psychology and Language Sciences University College London London UK; ^2^ Dyscover Leatherhead UK; ^3^ Centre for Health Economics and Medicines Evaluation (CHEME) Bangor University Wales UK; ^4^ The Aphasia Center at Jefferson Moss‐Magee Rehabilitation Jefferson Moss Rehabilitation Research Institute Philadelphia USA; ^5^ Istituti Clinici Scientifici Maugeri IRCCS, Laboratory of Neuropsychology Institute of Bari Bari Italy; ^6^ Cognitive Psychology and Neuropsychology Department University of Mons Mons Belgium; ^7^ Department of Medicine University of Washington Seattle Washington USA; ^8^ Department of Speech, Language, and Hearing Sciences University of Texas‐Austin Austin Texas USA; ^9^ Royal College of Speech and Language Therapy London UK; ^10^ Department of Speech and Language Therapy University of the Peloponnese Kalamata Greece; ^11^ Memory and Aphasia Care Chicago USA; ^12^ Research Chair on Primary Progressive Aphasia–Fondation de la famille Lemaire CHU de Québec‐Université Laval Quebec Canada; ^13^ Department of Hearing and Speech Sciences Vanderbilt University Nashville Tennessee USA; ^14^ Taylor‐Rubin Speech Language Pathology Sydney Australia; ^15^ Dementia Research Centre, UCL Queen Square Institute of Neurology University College London London UK; ^16^ Rare Dementia Support, Dementia Research Centre, UCL Queen Square Institute of Neurology University College London London UK

**Keywords:** awareness campaign, patient and public involvement, primary progressive aphasia, speech and language therapy, speech pathology

## Abstract

**Introduction:**

Globally, there is a lack of awareness of rare dementias such as those led by language decline (the primary progressive aphasias, PPA). The main treatment for PPA is speech and language therapy, yet many people are unaware of the benefits and are often not referred on by other health care professionals. Delays in diagnosis can exacerbate a delay in accessing resources such as speech and language therapy which can assist in maintenance of communication and independence. Some people affected by PPA are able to seek out information independently and navigate their own way to these services, but others experience substantial challenges in accessing support. This study was born out of discussions with people affected by PPA who identified an urgent need to raise awareness of PPA and the benefits of speech and language therapy. The aim of the project was to co‐develop a PPA awareness campaign and, through feedback, understand the impact of a co‐produced and delivered awareness campaign on awareness and knowledge of PPA and the role of the speech and language therapy.

**Methods:**

This study was informed by the People with Aphasia and Other Layperson Involvement (PAOLI) framework for guiding patient and public involvement (PPI) in aphasia research. Key components of the PPA awareness campaign were co‐developed at a World Café event with 30 people affected by PPA. Consequently, engagement data were collected from social media posts during the 10‐week campaign, and registration and attendance at the co‐planned webinars. A feedback survey was collected from attendees after the event, and a micro‐costing analysis conducted to understand the costs.

**Results:**

The 10‐week PPA awareness campaign focused on raising awareness of the role of speech and language therapy for PPA and a bespoke logo was developed that was shared across seven participating countries internationally. Attendees reported increased knowledge of the role of speech and language therapists and valued hearing the voices of people affected by PPA throughout the campaign. Webinar events were well attended across the participating countries with an average of 300 attendees, ranging from 233 in Greece to 369 in the UK. The cost of developing the awareness campaign was calculated at £26.20 per webinar attendee in the UK.

**Discussion:**

The PPA awareness campaign was conceived by people affected by PPA and aimed to increase awareness that speech and language therapy can support people affected by the condition. The awareness campaign developed iteratively and concluded with an awareness day event spanning seven‐countries internationally which are all now permanently accessible as resources on the International Speech and Language Therapist / Pathologist PPA network (International SLT/P PPA network) website. Future campaigns will take a more focused approach by targeting more specific audiences such as trainee doctors. The co‐development blueprint for the PPA awareness campaigns provides actionable, field‐tested recommendations for planning future health awareness initiatives.

**WHAT THIS PAPER ADDS:**

- *What is already known on the subject*
  Primary Progressive Aphasia (PPA) is a rare language led dementia for which speech and language therapy is the main treatment. At present, people affected by PPA report a lack of information available about speech and language therapy. This can perpetuate feelings of loneliness and social isolation.
- *What this paper adds to the existing knowledge*
  This paper provides the key components of a co‐developed PPA awareness campaign and describes a collaboration across organisations and countries to deliver the first year of the PPA awareness campaign. Future PPA awareness campaigns will build on this, by targeting specific audiences and aiming to increase knowledge and awareness of the role of the speech and language therapist.
- *What are the clinical implications of this study?*
  Increasing awareness of PPA and the role of the speech and language therapist has been identified by people with lived experience in the UK, as the core care pathway for people with PPA and their families. The PPA awareness campaign has made this information available to people affected by PPA and health care professionals. This includes a set of recommendations for the development of future similar awareness campaigns beyond PPA.

## 1 Introduction

The global prevalence of dementia is rising, with approximately 48 million people currently affected, and projected to reach 131 million by 2050 (Wolters and Ikram 2018). Around one million of these cases are in the UK (Alzheimer's Research UK 2024). However, members of the public are often unaware that dementia is an umbrella term for a number of different progressive neurological conditions which have in common their impact on independence in carrying out activities of daily living due to changes in cognitive functioning including memory, language, behaviour and visual perceptual skills (World Health Organisation 2025). The most recognised type of dementia by members of the public is a memory led dementia, generally associated with typical Alzheimer's disease (Stites et al. 2018) and which accounts for around 60%–70% of people with dementia worldwide (World Health Organisation 2025).

Rarer dementias such as primary progressive aphasia (PPA) are less well known compared to Alzheimer's disease, by both members of the public and health care professionals. Rare dementias collectively account for around 15% of dementia cases (National Institute for Health Research 2024) and are often misdiagnosed or diagnosed 2–5 years later than more typical dementia syndromes (Giebel et al. 2024). PPA describes a group of rare language led dementias, with conservative estimates suggesting that around 3,000 people are living with this diagnosis in the UK (Coyle‐Gilchrist et al. 2016). This rare language led dementia affects younger as well as older people but can have disproportionate and catastrophic impacts on working, social and family life because it disrupts communication and connection with others (Hardy et al. 2024a; Hardy et al. 2024b). There are three internationally recognised PPA variants with distinct language profiles and associated pathophysiology (Gorno‐Tempini et al. 2011; Rukensaite et al. 2021). Non‐fluent/agrammatic PPA (nfvPPA) is associated with an underlying frontotemporal pathology and causes difficulties in speech co‐ordination and articulation (termed apraxia) and/or using and understanding grammar (agrammatism). The semantic variant of PPA (svPPA) is also associated with a frontotemporal pathology but causes a gradual dissolution of word meanings affecting both word comprehension and naming. Over time people with svPPA develop more difficulties with non‐verbal object knowledge and socio‐emotional behaviour (Patterson et al. 2007). Logopenic PPA (lvPPA) is generally associated with an underlying Alzheimer's pathology and people with the diagnosis experience difficulties in word retrieval that have been attributed to an impairment of phonological processing/phonological working memory. People with lvPPA are considered to have an impairment of verbal memory which results in difficulties in comprehension of longer verbally presented information and word finding difficulties characterised by phonemic errors and paraphasias. Importantly, between 15% and 30% of people with PPA are considered to present with a mixed variant, whereby their symptoms do not clearly fit into one single syndrome, instead people experience a mixture of symptoms from different PPA variants (Teichmann 2021). Though presenting initially as a language led dementia, people with PPA progress inexorably over time and can also develop cognitive difficulties such as apathy (nfvPPA), sequencing difficulties that is, knowing what order to do things such as making a cup of tea or getting dressed (lvPPA) and a lack of empathy (svPPA) (Belder et al. 2024; Foxe et al. 2021).

The main treatment for PPA is speech and language therapy (Taylor‐Rubin et al. 2021). A range of speech and language interventions have been developed (Wauters et al. 2024) that aim to address areas that people with PPA and their families wish to change about their lives with PPA such as support for “talking to people” and “having conversations with families and friends” (Volkmer et al. 2025a). Research studies have demonstrated positive benefits including maintenance of access to personally relevant words (lexical retrieval interventions) (Croot 2018), maintenance of fluency and grammatical well‐formedness (script training) (Henry et al. 2018), and support for people with PPA and their family and friends to have better conversations (communication partner training) (Volkmer et al. 2023). These interventions can be beneficial when delivered individually or in groups (Watanabe et al. 2024) and the impact of some interventions has been shown to traverse languages for multilingual people with PPA (Grasso et al. 2021). Importantly, research has demonstrated that commencing speech and language interventions earlier has been shown to result in more (Wood et al. 2016), and longer lasting benefits (Cardorio et al. 2017) on communication and quality of life (Gauch et al. 2025).

A lack of awareness that speech and language therapy can support people with PPA amongst referrers can be a barrier to accessing timely support. Given speech and language therapy builds on preserved capacities to compensate for deficits, it is important that a person is referred as early as possible, while they still have several preserved cognitive abilities (e.g., episodic memory and semantic memory to support learning). Speech and language therapists (SLTs) in the UK report people with PPA are often referred too late to fully benefit from their services (Volkmer et al. 2020). This has also been reported in several other countries including Italy (Battista et al. 2023); Australia (Taylor et al. 2009); Germany (Gauch et al. 2026) and Turkey (Yaşa 2023). More recently, global surveys of SLTs working with people with PPA have highlighted that timing of referrals is influenced by potential referrers who are often unaware of the breadth of the role of SLTs with people with PPA (Gallée et al. 2024). In some countries it is possible that referrers perceive assessments of swallow safety and the later stages of the disease as the only useful service that SLTs are able to offer. SLTs internationally have expressed concerns that they “are not being given credibility” by their colleagues, meaning that they have to convince their colleagues of their role in helping people with PPA (Gallée et al. 2024). This is also reflected in detrimental information that might be conveyed, for example, “there's no point” in doing speech and language therapy (Ho et al. 2023).

People with PPA and their families themselves have reported a lack of knowledge of the role of the SLT (Loizidou et al. 2023). Concerningly, a lack of appropriate post‐diagnostic support can lead to feelings of confusion, frustration, and prolonged uncertainty for people with PPA (Davies and Howe 2020). Where people have been able to access information, often via their own research or via personal networks, this has on occasion enabled them or their families to navigate to services. Unfortunately, in some cases, even if they do know about speech and language therapy, people with PPA and their families have described challenges in locating health care professionals who understand their condition and how to support their needs (Beales et al. 2019; Davies and Howe 2020). People with PPA and their families have emphasised an absence of guidance and information as barriers to accessing speech and language therapy (Ho et al. 2023). This highlights an urgent need to increase accessible care pathways that point health care professionals and people with PPA and their families to speech and language therapy.

Increasing awareness of PPA and the benefits of speech and language therapy has the potential to increase access to support for people affected by the diagnosis. Internationally, awareness of other communication disorders such as stroke‐related aphasia also remains low (Hill et al. 2019). However local studies in the UK have demonstrated increased awareness over time, which the researchers attribute to increased presence of aphasia in the media, and work undertaken by local charities to raise awareness (Hill et al. 2019). Indeed, when consulted on future awareness campaigns, people with stroke‐related aphasia have emphasised the importance of increasing awareness to dispel misconceptions and stereotypes of communication difficulties (Bennington et al. 2025). Therefore, an awareness campaign for people affected by PPA should be co‐developed by people with lived experience to ensure it addresses what is important to them.

Health awareness campaigns such as awareness days and months have increased in popularity, with more than 300 health related awareness days or weeks every year (https://www.awarenessdays.com/awareness‐days‐calendar/category/health‐wellbeing/). Whilst the ultimate aim of awareness days is to improve health behaviours (such as visiting a General Practitioner (GP) to seek referral to a SLT), there is a lack of research evidencing improved health outcomes or changes in health‐related behaviours as a result of this type of campaign (Vernon et al. 2021). A recent systematic review demonstrated that awareness days can, however, improve information seeking behaviours and knowledge levels (Vernon et al. 2021), though the review cautions that there is little evidence of whether the costs involved are worth the outcomes. They recommend that researchers and policymakers should refrain from proposing health awareness days, weeks, and months as a solution to improving a particular health issue unless there exists specific, evidence‐based research to support it. Importantly, researchers have also warned of a lack of resources and experience as barriers to campaign success (Bennington et al. 2024). Successful collaboration with the right organisation, however, (such as government organisations, professional bodies, charities or libraries) can increase trustworthiness and reach of an awareness campaign (Rhode 2023).

The current study was inspired by proposals from people with PPA and their families who emphasised that alongside the research on PPA interventions, there was an important requirement for a single key message—increasing awareness of PPA and the need for speech and language therapy. Given the evidence that awareness campaigns can improve knowledge and dispel misconceptions, authors AV and RT collaborated with people affected by PPA and the Royal College of Speech and Language Therapists to co‐develop a PPA awareness campaign. Including people affected by PPA from the outset was vital to ensure that the work was relevant and useful (NIHR 2026). Researchers have found it challenging to include people affected by communication difficulties in research (Charalambous et al. 2023). Given the goal of including people affected by PPA from the start, this study was informed by the People with Aphasia and Other Layperson Involvement (PAOLI) framework for guiding patient and public involvement (PPI) in aphasia research (Charalambous et al. 2023).

With the ultimate goal of increasing awareness that speech and language therapy can support people with PPA, this co‐produced awareness campaign aimed to:

- Co‐produce the key components of an awareness campaign focusing on speech and language therapy for people with PPA and their care partners
- Evaluate engagement with the PPA awareness campaign
- Through feedback, understand the impact of a co‐produced and delivered awareness campaign on awareness and knowledge of PPA and the role of the SLT
- Characterise the micro‐costing of the PPA awareness campaign

## 2 Methods

**FIGURE 1:**
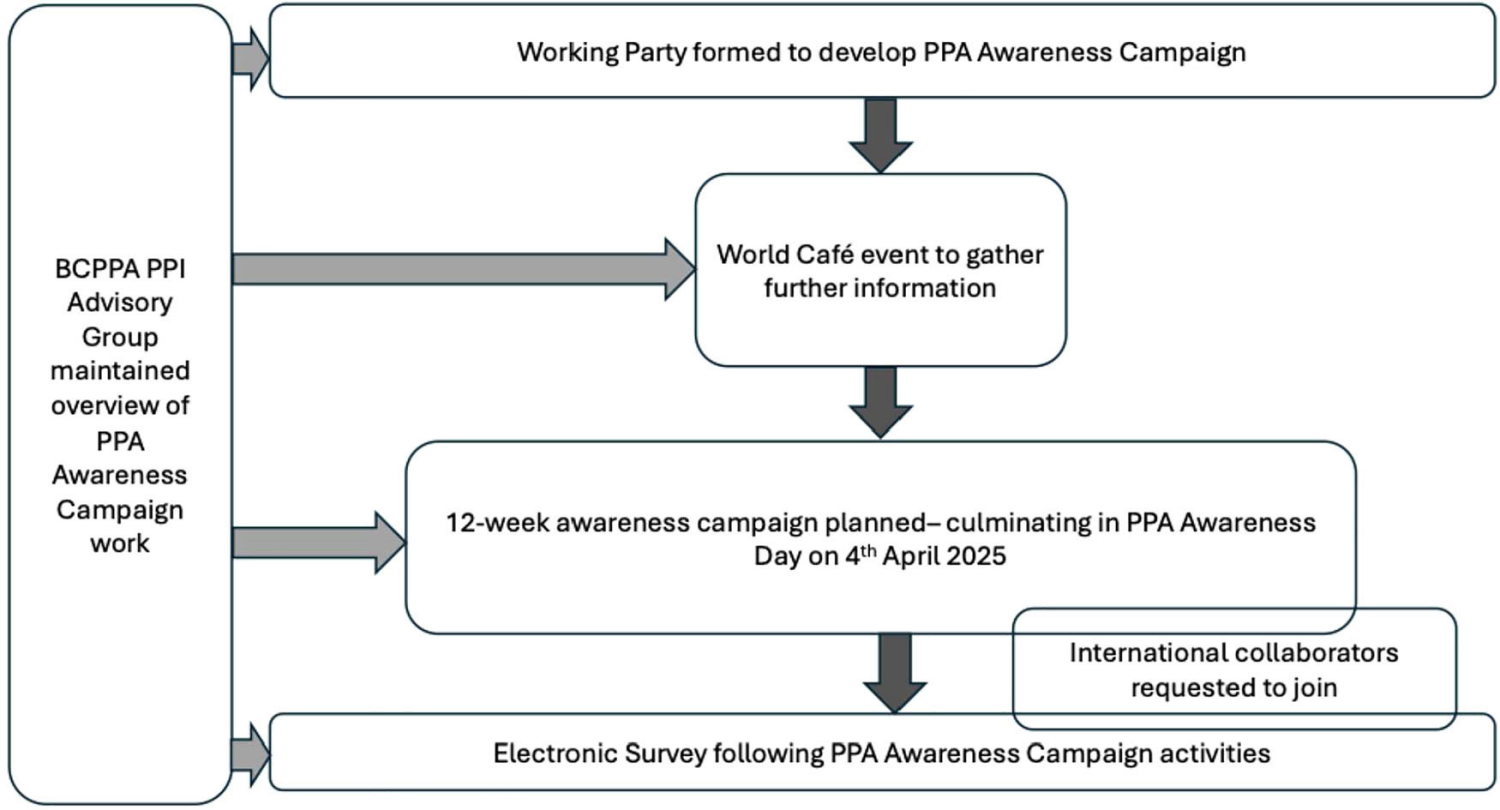
Co‐production of the PPA awareness campaign.

Guided by the PAOLI framework for guiding patient and public involvement (PPI) in aphasia research (Charalambous et al. 2023), this study used participatory methods to co‐develop the PPA awareness campaign. Figure 1 provides an overview of activities undertaken during the PPA awareness campaign. The Better Conversations with PPA PPI advisory group, comprising five couples, where one person had a diagnosis of PPA and their partners, participated in every stage of planning and analysing the results of the study and are represented as co‐authors on this paper. World Café methods were used to generate ideas from a wider group of people affected by PPA to inform the key components of the PPA awareness work. The PPA awareness campaign was co‐produced by the Better Conversations with PPA (BCPPA) PPI group, with involvement of other key stakeholders.

The study was a co‐produced work, as defined by the National Institute for Health and Care Research (NIHR)—“an approach in which researchers, practitioners and the public work together, sharing power and responsibility from the start to the end of the project” (NIHR website, accessed January 2025). Importantly, the NIHR flags that co‐production includes all necessary views and perspectives and given their status as members of the public working together with researchers, no personal data was collected from people affected by PPA. This work therefore qualified as PPI, meaning that at that time no ethical approval was required or sought.

### 2.1 Procedures

Whilst the PAOLI framework was developed for involvement of people with non‐progressive aphasia, the research team felt the model to be relevant to all those affected by PPA, including care partners, as it provides a tool to equalise power dynamics and maximise engagement of people regardless of level of communication, education and other linguistic or social disparities. This is consistent with the ethics of the BCPPA PPI advisory group, who have advocated for using the same accessible information and strategies in all aspects of our research work. The authors recognise that the PAOLI framework does not make provision for people living with progressive aphasia, and additional considerations made in addition to those outlined in framework are flagged throughout the methods section. The PAOLI framework outlines four phases:

#### 2.1.1 Phase 1: Foundation

The BCPPA PPI advisory group was formed in late 2022 by the lead author (AV), SLT and researcher, working on the BCPPA and rare dementia NHS embedded randomised waitlist controlled trial (trial registration number ISRCTN16268666; https://doi.org/10.1186/ISRCTN16268666). Group members were sent an email invitation circulated to members of the Rare Dementia Support Group (https://www.raredementiasupport.org/) and other support networks including Dyscover (https://dyscover.org.uk/) and a Manchester Frontotemporal dementia support group known to AV. Following several enquires the BCPPA PPI advisory group was finalised once the first five couples, where one person had PPA and an invited communication partner, decided they would like to become members of the group. The five dyads represented two people with lvPPA, two people with nfvPPA and one with mixed PPA and their spouses or life partners. The idea of a PPA awareness campaign was driven by the BCPPA PPI advisory group and other people with PPA and their families, who co‐produced the seven care pathway recommendations for PPA with the lead author (Volkmer et al. 2025b). The first and top recommendation was to raise awareness and understanding of PPA and the role of speech and language therapy. On the recommendation of the BCPPA PPI advisory group, several additional key stakeholders were consulted for advice about increasing awareness of PPA including undertaking wider consultation with a more representative group of people affected by PPA from across the UK, speaking to the Royal College of Speech and Language Therapists (RCSLT); key third sector organisations involved in providing speech and language therapy for people with PPA—Dyscover; and support for people with PPA—Rare Dementia Support (RDS). From these a working party was formed to lead the development of a PPA awareness campaign, comprising the lead author AV in her role as a researcher in the field of PPA, and representatives from each of the above organisations (RT from Dyscover, PJ from RCSLT, NZ from RDS) and PR on behalf of people affected by PPA.

#### 2.1.2 Phase 2: Development

**FIGURE 2:**
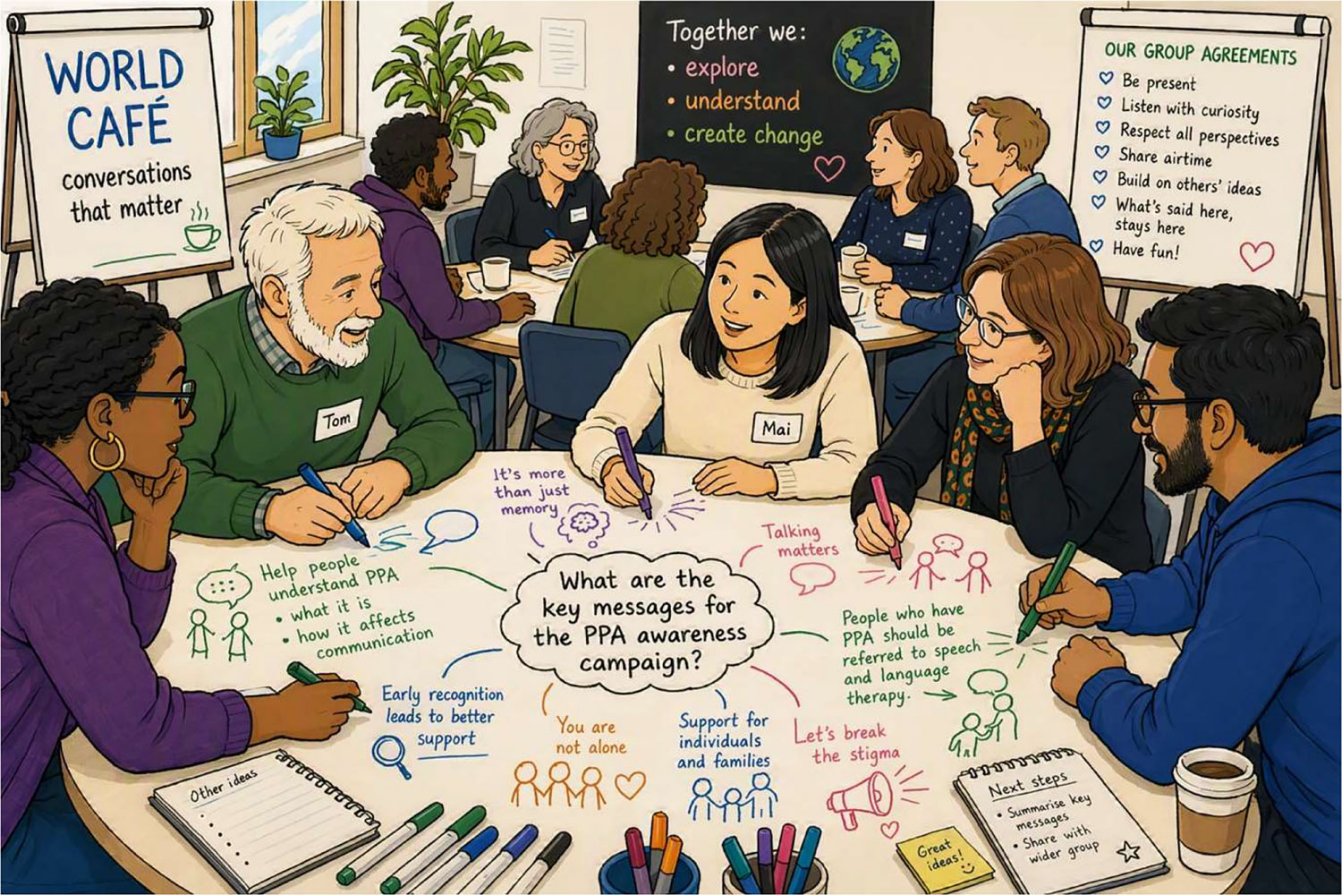
Illustration of a World Café co‐production workshop exploring key messages for a primary progressive aphasia (PPA) awareness campaign. The image is an AI‐generated illustration created using Microsoft Copilot (2026).

Developing ideas for the awareness campaign: In order to gather information from a wider group of people with lived experience on what the PPA awareness campaign should include, a World Café task methodology (Löhr et al. 2020) was employed. The World Café was held at a large PPA event with 30 attendees with PPA and their family care partners. The 2‐hour PPA event is held annually by the lead author AV, in collaboration RT, a SLT and service lead at Dyscover. Information about the event is disseminated via the charity's mailing list and clinical networks, inviting people to respond to RT if they wish to attend. The event enables people with PPA and their families to come together for peer support and to hear about different aspects of current speech and language therapy practice. A World Café methodology was selected because it provided a structured and creative setting that encouraged people affected by PPA to share ideas, as described in previous qualitative co‐production studies with people with dementia and PPA (Bates et al. 2022). The method promotes open discussion and equal relationships to maximise participation and power imbalances. The lead author has previously also used World Café methods and embedded supported communication techniques to maximise communication participation (Volkmer et al. 2025b). Five elements of the World Café method (Bates et al. 2022) were considered including 1. Setting—attendees were invited to sit around café style tables, set up around an open room in the University, 2. Welcome and introduction—attendees were welcomed to the event by AV and RT and invited to participate in a range of activities of their choosing including the World Café work to develop the awareness campaign led by AV and an artist colleague MR, or supported social networking, led by RT and colleagues; 3. Small group rounds—attendees were invited to form small groups around four tables, where they were provided with coloured pens, pencils and large pieces of paper and invited to note down or draw ideas. The first author AV provided support for communication, occasionally facilitating the discussion by re‐explaining the task using simplified language, writing key words or acting as scribe when participants identified ideas. MH also acted as scribe drawing ideas as attendees described them. Members of the BCPPA PPI advisory group participated in the World Café event by spreading themselves across the tables and providing additional opportunities for other attendees to clarify questions and share ideas related to the agenda. Figure 2 depicts a World Café co‐participatory research activity used to identify key messages for the PPA awareness campaign. Once attendees had been at a table for around 20 minutes they were encouraged to move onto the next table. 4. Questions (decided by the working party)—Participants were asked to respond to four main questions—What should a PPA awareness logo include? Who are the audience to be targeted for the awareness campaign? What are the key messages for the awareness campaign? and What is the timeline for the awareness campaign? 5. Harvest—results of the World Café event were presented to the wider group immediately at the end of the meeting by AV, using accessible communication, specifically a photo summary from written and drawn information, alongside key words, emphasis and gesture. The summary was also presented to the BCPPA PPI advisory group for further feedback, and consequently to the campaign working parting.

PPA awareness campaign: The working party used the generated ideas to inform the co‐production of a 10‐week awareness campaign starting on Friday 31st January 2025, culminating in a PPA awareness day webinar on Friday 4th April. The awareness campaign leveraged expertise from all working party members to address the identified audiences. As an example, the charity that RT worked for invited their local Member of Parliament (MP) to visit their premises and asked the MP to host a parliamentary event at the Houses of Parliament. As chair of the International Speech and Language Therapist / Pathologist PPA network (International SLT/P PPA network), and co‐lead of the UK SLT PPA network the lead author disseminated information to speech and language therapists/pathologists internationally and identified collaborators to host potential podcasts and social media activities. During the initial phase of the campaign, after AV and RT had shared news of the planned awareness campaign with the International SLT/P PPA Network they are members of, an international collaborator (ML) approached the working party asking if they could join the campaign. AV and RT opened the campaign to all members of the International SLT/P PPA Network and further members joined including MH, SG, JG, SA, BK, KX, NK, PB & SB. AV and RT shared the logo, planned structure of the PPA day webinar and access to all produced resources with these collaborators who independently designed awareness day webinars in their respective countries across Australia, Canada, France (intended for French, Belgian and Swiss audiences), Greece, Italy and the United States of America.

AV and RT led the dissemination of planned awareness day activities in the UK every Friday for 10 weeks commencing 31st January 2025. Information related to the campaign was disseminated via an email to AV and RT's networks on the first Friday, inviting addressees to sign up to weekly emails. This was also disseminated by NZ to the RDS PPA Support Group members. Those who did respond to sign up received a weekly email for the duration of the campaign. AV and RT established a dedicated social media account on the Bluesky platform and leads AV and RT used their professional social media accounts across X, Bluesky and LinkedIn to further disseminate information. All posts were disseminated with a dedicated hashtag #PPAawareness every Friday. On 4th April 2025, the date of the PPA Awareness Day, live webinars were held across six participating countries including Canada, France, Greece, Italy, UK and USA. The webinars generally comprised a presentation by a SLT, a neurologists/neuropsychologist, a support or charity organisation and people with lived experience from each of the participating countries. The webinars were recorded and are now hosted in perpetuity on the International SLT/P PPA network website: https://speechtherapyppa.com/ppa‐awareness‐day. News events were also disseminated across local news outlets in Italy, New Zealand and Norway. The Australian event took place 6 months later in September 2025, to coincide with Dementia Action Week in that country.

#### 2.1.3 Phase 3: Translational

Engagement data: To evaluate the impact of the PPA awareness campaign the working party collated data related to each of the outputs. Specifically, engagement with all social media posts and videos was examined and data on total views on 25th May 2025 collected (and updated on November 5th, prior to submission for publication), and the number of webinar registrants and attendees was counted across all participating countries.

##### 2.1.3.1 Feedback

An anonymous feedback survey (see Appendix 1), hosted on the Microsoft Forms platform, was disseminated via social media, to a list of email recipients who had signed up for weekly campaign updates and to UK based webinar registrants. The UK based survey comprised seven questions (designed by the research team) which aimed to capture feedback on this year's awareness campaign, and opinions to inform future awareness campaigns. Responses were completely anonymous and no demographic information was collected. Responses have been analysed using descriptive statistics and open responses were independently coded by two researchers (AV and RT) using “conventional content analysis” (Hsieh and Shannon 2005). This approach was felt to be most relevant given the brevity of the responses, and the focus on capturing patterns and opinions of participants to inform future awareness campaigns. Content analysis allows for the collection and summarizing of data, without immersing or exploring the latent meanings (Hsieh and Shannon 2005). AV and RT independently coded data, AV compared coding and synthesised a brief narrative summary. Results were presented to people with PPA and their families in the BCPPA PPI advisory group and at a consequent annual PPA event held by AV and RT (as described previously). This member checking approach ensured the work to develop and evaluate the PPA awareness campaign remained grounded in the experiences of people affected by PPA. This also enhanced the reflexivity in coding of data by AV and RT, who had both led the awareness campaign activities and may be considered to have been overly invested in the event. A narrative synthesis of the responses to open questions is reported in the results of this manuscript, and the discussion is informed by the discussion of the data with people affected by PPA. Webinar hosts in Greece (NK), France (SB) and Australia (CTR) collected similar feedback data from webinar attendees who shared a brief narrative summary of their results with AV and RT (this data was not included in the analysis of the UK data).

##### 2.1.3.2 Micro‐Costing for UK Campaign

We also conducted a micro‐costing analysis from a provider perspective to explore the direct costs of the awareness campaign including staff time and material costs. The provider perspective included all upfront costs borne by the employers of the two project leads (AV and RT) a university and a third sector charitable organisation. A bottom‐up micro‐costing approach was used as it allowed for collection of detailed resource use data allowing accurate costing of the awareness campaign. The two leads completed cost diaries for the campaign (see Appendix 2), which included time spent in meetings to plan and prepare activities, time spent writing materials such as slides, and time spent delivering the webinar. Time was costed using AV and RT's hourly salary rate including on‐costs. Additional costs included travel costs, room hire, photographer and catering for the parliament event and honorariums for the PPI advisory group meetings. Costs were presented in British Pounds Sterling (£) for the cost year 2024/5. This base case analysis explored the upfront costs of delivering the awareness campaign. Given the focus of the awareness day itself was a webinar, a sensitivity analysis was also conducted by calculating additional time spent by contributors (Consultant Neurologist, an additional SLT, representative from Rare Dementia Support and additional PPI presenters) to explore potential extra costs for future similar webinars. Costs were derived from the most recent Personal Social Services Research Unit (PSSRU) publication of unit costs (Jones et al. 2025) and the NIHR guidelines for PPI reimbursement which includes honorariums, travel costs and meeting refreshments (see Appendix 2 for further information on these).

#### 2.1.4 Phase 4: Ongoing Processes

Plans for succession to maintain the PPA Awareness Day into the future are also discussed in this paper. Given the lack of guidance available on how to develop awareness campaigns about communication disorders the authors have synthesised a set of guidance recommendations for future similar campaigns, which is reported in the discussion of the current paper. These recommendations were synthesised by authors AV and RT, based on debriefs and discussions following the awareness campaign. Recommendations were then shared with co‐authors for refinements (particularly wording of suggestions).

Whilst the PAOLI framework is designed for people with non‐progressive aphasia (Charalambous et al. 2023), this study reports on extending this to those affected by PPA and their care partners. The BCPPA PPI advisory group have reflected with the authors on several occasions about how to address the evolving needs of people with progressive aphasia, specifically as group members have felt less able to participate or attend group meetings. Negotiating withdrawal from PPI activities requires planning from the outset, not just for those members and their partners who may wish to withdraw, but for the remaining members who will likely feel concerned about the ongoing physical and mental well‐being of people they have developed a close relationship. This distinction also means that the core group of participants changes over time, and methodology for engagement must therefore have an element of succession planning for any advisory group membership. Over the course of the current study, the BCPPA PPI advisory group membership did change. Of the original six couples, two chose to step back from their roles, and two new couples joined the group. Navigating a progressive condition whilst undertaking PPI work, requires frequent recalibration and emotional support for all members. This is an important addition for future PPI frameworks.

## 3 Results

### 3.1 Components of the PPA Awareness Campaign

A narrative summary of ideas generated from the World Café event addressing the four key questions are summarised in Table 1. The working party “harvested” the World Café data and agreed, through discussion, on outcomes, which are also presented in Table 1. The working party used the collated information to synthesise a logo by emailing the accessible photo summary from the World Café event to a graphic designer. The graphic designer produced an initial draft, which was circulated to the working party and wider BCPPA PPI advisory group for comments, and feedback on the colours, texture and alignment of the logo returned for final refinements. Participants agreed on the logo “Primary Progressive Aphasia—A language led dementia”, and no‐one raised concerns about the term ‘dementia’ as stigmatising, rather that it would dispel myths around the condition. Consequently, discussion for logo refinements focused on colour and word alignment. This final logo is presented in Table 1, alongside a summary of other outcomes from the World Café event.

**TABLE 1:** Summary of discussions from the World Café event addressing four key questions.

Four main questions	Narrative summary of key comments	Outcome from harvesting of World Café in the project working party
What should a PPA awareness logo include?	It should include the diagnosis name and be usable for campaigns beyond the first (durable). It should express a gradual loss for example, through the colour/texture of the font. It could include an open door/image but this is not essential.	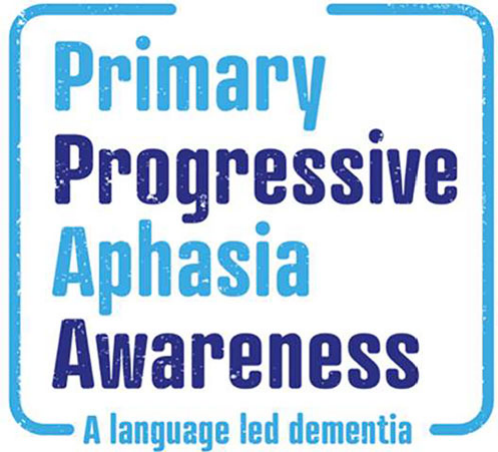 (designed by Jempomak.com)
Who are the audience to be targeted for the awareness campaign?	Ultimately—the wider public including large organisations and politicians who make decisions about healthcare funding.	Whilst all agreed that this was highly ambitious, attendees felt the following were crucial, but we should aspire to be aspirational for the future. Crucial audience: People living with PPA People who might have PPA Their families and friends Health care professionals (people who might refer to speech and language therapy)
What are the key messages for the awareness campaign?	Discussion centred around advocating for increased funding of speech and language therapy services for people with PPA and their families and influencing government organisations and politicians to prioritise funding for people with rare dementias. The topic of public awareness to enable people to seek diagnosis was also raised. Increasing awareness amongst organisations such as supermarkets and the wider community about speech, language and communication needs of people with PPA and how they can help people was identified as important.	The most important message for the 2025 awareness campaign was identified as: People who have PPA have significant communication difficulties for which they should be referred to speech and language therapy.
What is the timeline for the awareness campaign?	The awareness campaign should culminate in a PPA Awareness Day. It must be held on a date that does not coincide with other events and festivals including stroke‐related aphasia awareness month, FTD awareness week or other dementia awareness events. It should be soon—within 9‐months of the World Café event, in order to maintain momentum.	Awareness Day to be held on Friday 4th April 2025, with several events to be planned on a weekly basis in the weeks leading up to the event

### 3.2 PPA Awareness Campaign Engagement Data

PPA Awareness Campaign activities commenced on 31st January 2025, culminating in the PPA Awareness Day webinar on 4th April 2025. A description of each activity is summarised in Table 2, alongside relevant engagement data including social media views, and registration numbers and attendees at the seven respective webinar events. Approximately six‐weeks after the campaign, on 25th May 2025 the social media post launching the PPA Awareness campaign on X was the most viewed of all campaign related social media posts and activities, generating over 3,300 views. The webinar events were well attended across the six participating countries with an average of 300 attendees, ranging from 233 in Greece to 369 in the UK.

**TABLE 2:** Summary of PPA awareness campaign and PPA awareness day activities and associated engagement data.

Date of PPA Awareness Activity	Description of activity	Data (correct as of 25th May 2025)
31st January 2025	Launch of the PPA awareness day campaign, inviting people to sign up to follow weekly activities.	X post viewed more than 3,300 times.
7th February 2025	Collaboration with a trusted support organisation—re‐release of a Rare Dementia Support lived experience interview	X post viewed more than 2,500 times. https://www.youtube.com/watch?v=7zkuSmpN1HM (4,221 views since October 2020‐May 2025
14th February 2025	Collaboration with a trusted research organisation—NIHR Dementia Research podcast	X post viewed more than 2,600 times. 209 views on youtube: https://www.youtube.com/watch?v = t2k2‐UhJjbU
21st February 2025	Collaboration with clinical social media output—Brainy Speech podcast	X post viewed more than 343 times. Downloaded 30 times from https://podcasts.apple.com/gb/podcast/the‐brainy‐speech‐therapist‐podcast/id1803924147
27th February 2025	Promotion of resources for SLTs working with people with PPA—Book launch	X post viewed more than 1,200 times.
7th March 2025	Amplifying the voices of key stakeholders—co‐produced photo montage	X post viewed more than 1,600 times.
14th March 2025	Amplifying the voices of key stakeholders—person with PPA shared their poetry, via a pre‐recording made by their niece, on living with PPA	X post viewed more than 563 times.
21st March 2025	Collaboration with trusted organisation for student SLTs—UCL Giving Voice podcast with members of the BCPPA PPI advisory group, AV and RT	X post viewed more than 1,000 times. https://open.spotify.com/episode/7×28xwP0DKqXzrSojBhHr6
28th March 2025	Lobbying at government level with a trusted organisation—event hosted by Royal College of Speech and Language Therapists at the Houses of Parliament to raise awareness.	10 parliamentarians attend the event. Since the event there have been 3 questions in the House. X post viewed more than 1,300 times.
4th April 2025	PPA Awareness Day—webinars across UK, USA, Canada, France, Italy and Greece.	X post viewed more than 2,400 times. https://speechtherapyppa.com/ppa‐awareness‐day.html Australia: 350 registered. To facilitate access, group hubs were encouraged. 42 attendees indicated that they were planning to organise their own hub to watch the webinar as a group so there were likely more people watching online who were not registered. Canada:250 registered to attend the webinar and 150 attendees France: 319 registered to attend the webinar Greece: 391 registered to attend the webinar, and 233 attended. Italy: 350 registered to attend the webinar, and 185 attended. UK: 702 people registered to attend the webinar and 369 attendees. USA: 101 attendees and successful in obtaining a mayoral declaration of International PPA Awareness Day in Philadelphia.

### 3.3 Feedback on the PPA Awareness Campaign

The electronic survey circulated by AV and RT from the UK was completed by 61 anonymised respondents. 61 respondents rated the PPA awareness day (10 = very good, 0 = not very good), with a mean of 9.2 (range 5–10), similarly 61 respondents rated the clarity of the PPA awareness day aim of increasing awareness that people with PPA benefit from speech and language therapy (10 = very clear, 0 = not very clear), with a mean of 9.4 (range 5–10).

Open responses almost unanimously indicated the webinar event was the highlight of the PPA awareness day, with just under half of respondents (30) emphasising the value of hearing from people living with PPA and their families in the webinar:

“When there was a consensus from those living with PPA and their families that SLT had delivered ‘communication therapy’ and was so much more than their original ‘traditional’ expectations of SLT”

Others also emphasised the range of presenters including the neurologist (15), SLT and support services (12) and the clear, succinct presentation styles (6) as the best aspect of the webinar:

“Just the lovely simple communication used throughout everyone's delivery and the message we can include everyone even though far too often it's much easier to leave people out of the conversation. And that it is of course about being human together”

Just under half (30) reported that the webinar expanded their knowledge of PPA and support available:

“To Know Facts About the Disease” and “Useful Strategies to Help Families Cope and Conversation Partner Training”

One respondent emphasised that “To know that there are people working towards more understanding of PPA” was an important highlight, while two respondents valued the “message to the government”.

Just under a quarter (12) of respondents reported a desire for more information at future PPA awareness days. A small number of respondents (4) suggested a need for a longer webinar, and just under a quarter (12) suggested wider coverage of the PPA awareness day across additional media platforms such as national newspapers, radio and television. Posters and advertising campaigns were suggested, with specific suggestions of placing posters or leaflets in health centres/GP surgeries or targeting medical trainees as part of the work. Referrers (e.g., GPs and medical professionals) were felt to be a particularly relevant group who could be more directly targeted in future campaigns

Several suggestions were also aspirational and highlight attendees’ motivation for the PPA Awareness campaign to collaborate with other organisations such as the DEEP network, other charity and volunteer organisations to undertake more research. Targeting of NHS funding bodies in the UK was advocated, such as national and local commissioning bodies. One respondent suggested asking for donations for future awareness work. A similarly creative suggestion promoted badges “to spark curiosity”.

There were a small number of technical comments, with three respondents requesting more written information about the event prior to the webinar. One respondent found the descriptions of living with PPA 9 years on to be “rather frightening” and another felt the event “came across as SLT promotion‐ the roles of other professionals were barely considered”. In fact, one respondent asked for more information in future webinars or events about other professional's role, more specific information about strategies or therapy they could use at home to support communication and opportunities to actually attend social networking or practice. A small number of respondents (4; presumably professionals) requested further sessions on how to deliver interventions.

Feedback from webinars hosted in other countries was similar to that from the UK event, with positive feedback about the inclusion of people with lived experience and no salient differences in the feedback across countries. The Greek language feedback form was completed by 29 respondents. Participants rated the event with a mean score of 9.55/10 (SD = 0.57) and the clarity of goals at 9.75/10 (SD = 0.44). Qualitative feedback emphasised the value of including the voices of individuals with PPA, and the importance of multidisciplinary presentations. Participants also suggested expanding media outreach and establishing an online community.

The French language feedback from was completed by 21 respondents who were very satisfied with the presentation. For next year, many people would like the advice for carers to be more detailed.

The Australian feedback form was completed by 21 respondents, who were very satisfied with the presentation.

### 3.4 Micro Costing for the UK

#### 3.4.1 Base Case Analysis

The total cost of delivering the PPA awareness campaign in the UK was £9,669.844. The largest driver of cost was time spent planning the PPA awareness day activities, writing materials and slides comprising £4087.10. Based on the total cost and number of webinar registrants (702) / attendees (369) this equates to £13.77 per registrant/£26.20 per webinar attendee (for a breakdown of costs see Appendix 2). A sensitivity analysis incorporating hypothetical costs for volunteered time to account for additional staff and PPI costs for the webinar added an additional £810.34 *to the total costs*.

## 4 Discussion

The aim of this work was to raise knowledge and awareness of PPA and the role of speech and language therapy through a co‐developed awareness campaign culminating in a PPA awareness day webinar on 4th April 2025. Feedback was overwhelmingly positive, indicating benefits to attendees such as improved knowledge of the breadth of the role of the SLT and a desire for more, similar, future events. Whilst no demographic data was collected from webinar registrants (702 people) or attendees (369 people) in the UK it is assumed a large proportion were those affected by the disease (based on comments in the feedback on the relevance of the webinar content), meaning a significant number of the 3000 people diagnosed with PPA (Coyle‐Gilchrist et al. 2016) were represented in some way (either by way of family and friends or themselves in attendance). Given the lack of social media data reported in the research literature about social media impact in other comparable awareness campaigns (whether stroke‐related aphasia or rare health conditions), it is difficult to know what qualifies as success here. The cost for this awareness campaign in the UK equates to £13.77 per registrant and £26.20 per attendee, but it is difficult to ascertain the true cost per view given the lack of data collected on the number of attendees at each screen and the number views of the recordings on the permanent website platform. Additionally, whilst we costed the time spent by the UK based leads (AV & RT) working with international collaborators, this does not represent the cost per number of global attendees, nor the costs of international collaborators.

Results from the survey indicate that the PPA awareness campaign, specifically the webinar event, improved attendees’ knowledge of PPA and the available supports, including speech and language therapy. When asked what the best thing about the PPA awareness day was, just under half (30) of respondents reported that the webinar expanded their knowledge of PPA and support available. Additionally, the number of interactions with social media outputs suggest the opportunities to access information about PPA enabled increased information seeking behaviour. These results are in line with previous research which has emphasised that awareness campaigns can improve information seeking behaviour, awareness and sometimes knowledge but there is little evidence that campaigns can change health management behaviours. Given dementia is now more feared than cancer amongst people in their middle age, there may be a genuine risk that raising awareness could also fuel “dementia worry” (Maxfield et al. 2024). PPI collaborators in the current study felt strongly that increasing understanding would reduce myths about PPA, and increase knowledge of speech and language therapy supports, which, ultimately, will improve quality of life for people living with the condition.

Unfortunately, we were unable to collect any information about the demographics of those who interacted with any aspects of the campaign. Based on feedback (e.g., comments on usefulness of strategies for families and requests for specific training in intervention delivery) it is likely that campaign captured primarily people affected by PPA and health care professionals within a close community for example, people who are linked in with SLTs and SLTs themselves. This may mean the PPA awareness campaign messages may have been largely received by people who were already informed on PPA and the role of SLT. Importantly, several respondents emphasised the value of interacting with people outside of this sphere such as politicians (through the Houses of Parliament event). Previous reviews of awareness campaigns have emphasised that the most successful ones target specific subpopulations such as specific health care professionals (Vernon et al. 2021). While this suggests the current campaign might have been somewhat diluted due to the lack of focus, it underlines recommendations from survey respondents suggesting that future awareness campaigns should prioritise (trainee) doctors, as the key referrers to speech and language therapy.

Survey responses following the UK PPA awareness campaign reported the value of hearing from people affected by PPA themselves as part of the campaign. Previous researchers have emphasised that health awareness days may be most useful for people affected by a condition, when they provide a sense of community (Rhode 2023). Failures in post‐diagnostic onward referral can have significant negative mental health outcomes for all people affected by PPA (Davies et al. 2025; Ho et al. 2023). People have reported feeling incrementally lonely and isolated with PPA, attributing this to both rapidly shrinking social networks in the context of a disease that impacts social skills, and living with a rare disease such as PPA (Pozzebon et al. 2018). Having people with lived experience of a rare communication difficulty as key campaign leaders and sharing their stories has been identified as a valued component of an awareness campaign (Bennington et al. 2024; Bennington et al. 2025). Importantly however, whilst the majority of feedback was positive, there was also some feedback highlighting the potential harm from awareness where that inevitably entails an awareness of suffering and debility. Therefore, alongside collaborating with trusted organisations, carefully curated testimonials from people affected by PPA, describing their experiences with speech and language therapy increased the trustworthiness of the information being shared as well as reducing the stigma of living with a rare progressive condition (Rhode 2023).

The PPA awareness campaign was conceived by people with PPA and their families inspired by a lack of awareness and available information on PPA and treatment and support options. In line with the international PPA Core Outcome Set, where almost two hundred people affected across 17 countries identified the importance of being able to talk and have conversations with family and friends (Volkmer et al. 2025a), people affected by PPA in this study identified the importance of being aware of treatments (speech and language therapy) that improve talking and support people to have better conversations. This awareness campaign was inspired by the community of people affected by PPA on the BCPPA PPI advisory group who were working towards co‐developing a care pathway. They emphasised the most important requirement for a single key message—increasing awareness of PPA and the need for speech and language therapy. Health care pathways, such as those produced by the National Institute for Care and Excellence (NICE 2018) are typically written by health and social care researchers, policy makers and professionals, transforming research evidence into specific recommendations. While they are increasing used by consumers‐ patients—often as a tool to advocate for their health care needs, the NICE guidance for dementia guidance, omits speech and language therapy despite increasing evidence of the benefits. In contrast, this PPA awareness campaign took a more inductive approach, grounded in the experiences of what people affected by PPA needed and identified as an information gap. Taking a collaborative approach enabled the synthesis of a key message situated in what people themselves felt was most important for their lives with PPA.

### 4.1 Strengths and Limitations

The strengths of the work to develop this awareness campaign are evidenced by the fact that it was inspired by, co‐developed, and led by people with PPA and their families. The PAOLI framework enabled the research team to keep people at the centre of the campaign throughout. However, this iterative approach to developing the campaign can be described as both its strength and limitation. Importantly, only the original BCPPA PPI advisors received any renumeration for their time—honorariums and travel costs. Unless all collaborators are paid the same, there remains an imbalance between research and participation. This is not without its own problems, given people with PPA and their families may not be able to access benefit payments if they are being paid a salary. The research community, funders and universities may need to address this more directly, to fully understand the tension and power imbalances in co‐produced research. Given the data collected across different countries differed and was differently analysed (only the UK feedback was analysed using content analysis), the international relevance of the results of this study are limited. The iterative nature of the campaign meant that the feedback survey did not directly ask about changes in awareness and knowledge, yet respondents commented on increased knowledge and awareness in response to other questions and only latterly were the limitations of having too broad an audience for the campaign considered as it made it harder to explore the type of knowledge gained, and no data on the actual audience demographics was collected from the UK event. This may have biased the webinar feedback, given respondents may not have been representative of the wider PPA spectrum. Understanding who engaged with the awareness campaign—which health care professionals/people affected by PPA, the geographical range, whether they had already seen a SLT of not—would have better informed the outcomes. If raised awareness is to lead to impactful action (more referrals to SLT) then the point of contact with healthcare professional for those with PPA/carers is where those who have no knowledge are going to be best captured. Additionally, the iterative nature of the campaign also meant we had limited data from international collaborators and their events. However, research on communities of practice has shown that organically grown, rather than didactically delivered networks lead to more relevant and genuine connections (James‐McAlpine et al. 2023). Thus, it is also important that the awareness campaign developed organically, ensuring its trustworthiness by keeping people affected by PPA at the centre. One limitation of co‐production is that we are unable to demonstrate the representativeness of the people affected by PPA who collaborated on the study. Notably, the BCPPA PPI advisory group did advocate for wider consultation with a range of more representative people affected by the condition. The same advisory group have also previously raised concerns that there is little representation from people from socio‐economically, linguistically and ethnically diverse communities within the wider PPA community who attend events and research‐based events. This has led to several studies exploring the experiences of accessing support for people with PPA and other rare dementias who are from these backgrounds (Volkmer et al. 2025c) which has highlighted that a lack of awareness and huge amounts of stigma within and outside their communities perpetuate barriers to accessing support. Future awareness campaigns should consider collaborating with cultural groups to enhance awareness amongst these specific communities.

### 4.2 Clinical Implications and Future Research

The success of the PPA awareness campaign depended on the collaborations with several trusted organisations who shared their knowledge and experience in supporting other similar campaigns in the past (e.g., RCSLT's knowledge of the dysarthria awareness day for example). For ongoing success and to maintain longevity local action is also required, and whilst the UK SLT PPA network supported the campaign future awareness campaigns should explore how to hand over to local speech and language therapy teams and empower them to influence upwards. The American Library Association have advocated for improved health literacy by producing tool kits and sharing resources to enable librarians to develop local campaigns more effectively (Rhode 2023). With this in mind, and to reduce cost implications for future awareness campaigns, the authors have synthesised a set of key recommendations for teams aiming to co‐develop future awareness campaigns:

*Synthesis of key recommendations for developing an awareness campaign*:

1. Deciding whether to host an awareness campaign should be undertaken with people with lived experience: It is possible that barriers to accessing support may be exacerbated by a lack of awareness of specific communication disorders. However, decisions to plan an awareness campaign are best identified ‘by’ and ‘with’ people rather the ‘for’ or ‘to’ people, therefore decisions about whether or not to plan an awareness campaign should be undertaken WITH a group of people affected by the condition.
2. Get advice from someone who has done this before: Find someone else who has done this before and ask for their advice, they may have useful contacts and resources available to save you time and energy.
3. Co‐produce an awareness campaign with a representative group: Identify your key stakeholders—people affected by a condition, and ensure you have a representative sample. This may mean a larger sample, or a sample representing a range of ethnic, linguistic and sociodemographic characteristics. This may also mean seeking advice across a specific or wide geographical area. Having brought together a group of people to plan an awareness campaign, make sure you form a core working party with additional stakeholders (representatives from trusted organisations).
4. Collaborate with trusted organisations: Collaborating with trusted organisations such as relevant universities, NHS and third sector organisations will improve the trustworthiness of your campaign.
5. Identify a target audience for the campaign: Identifying a target audience for the campaign, this makes it easier to understand what the key message is and how you measure change in your recipients of the message. It may be that a series of awareness campaigns are required as part of a wider campaign.
6. Your key message and aims: It is difficult to evidence whether awareness campaigns change health related behaviours, but they can improve awareness and knowledge, therefore tailor your aim with this in mind. Consider your brand, ensure your campaign is visible to the right people. Ask yourself Do you need a logo? Use social media, email, newsletters, podcasts, webinars, videos, radio, newspapers and any resources available within your resources.
7. Organise your time and resources in advance: Set yourself a timeline and stick to it. If you have any funds, if you do, plan this carefully. If you need funds, identify where they might come from. If you do not have funds, set boundaries with the co‐production group (without funds some things may not be possible).
8. Ask for help and build in a succession plan: Approach people who may be able to help, and accept offers of help when they come, you'll be surprised how many people want to help. The first year of the awareness campaign may only be the beginning. Build in a succession plan and find out what who will help you in the future. By sharing out the responsibilities a campaign is more likely to achieve longevity.

## 5 Conclusions

The PPA awareness campaign was conceived by people affected by PPA and aimed to increase awareness of the diagnosis and that speech and language therapy can support people affected by the condition. The awareness campaign developed iteratively and concluded with an awareness day event spanning six‐countries internationally. Feedback from the campaign demonstrated that the campaign improved awareness and a sense of community. Future campaigns should take a more focused approach by targeting more specific audiences such as trainee doctors, neurologists and regional SLTs. The experiences of the team who co‐developed the PPA awareness campaign provide a resource of recommendations for anyone planning future awareness campaigns.

## Funding

Anna Volkmer is funded by an NIHR Advanced Fellowship award NIHR: 302240. Jason D. Warren has received grant support from the Alzheimer's Society, the Royal National Institute for Deaf People, Alzheimer’s Research UK, the National Institute for Health Research University College London Hospitals Biomedical Research Centre and a Frontotemporal Dementia Research Studentship in Memory of David Blechner (funded through the National Brain Appeal).

## Conflicts of Interest

The authors declare no conflicts of interest.

## Supporting information




**Supporting File**: jlcd70324‐sup‐0001‐SuppMat.docx

## Data Availability

Data is available on request from the authors.
