# Supplementary material for: ‘It Is of Course About Being Human Together’: Co‐Development of a Primary Progressive Aphasia Awareness Campaign to Enhance Understanding of Speech and Language Therapy
Source: Int J Lang Commun Disord. 2026 Sep 8;61(5):e70324. doi: 10.1111/1460-6984.70324 (PMC13551054; doi:10.1111/1460-6984.70324)
Supplement: Supplementary file 1 [file JLCD-61-0-s001.docx]

**Appendices 1: Feedback Survey hosted on Microsoft forms**

Thank you for being part of the first ever International PPA awareness day.

We would like to collect your opinions and views on the first International PPA awareness day. We would really like to hear what you think so we can do better next year. 
Our plan is to continue having the international awareness day every year and we want to make sure it is an event that is based on what people actually find interesting, useful and inspiring.
Thank you for your time!

Anna and Rosemary

1. How would you rate the first ever International PPA awareness day?
   (1= not so good, 10= very good)
2. What was the best thing about the PPA awareness day in your opinion?
3. What was not so good or could have been done better about the PPA awareness day in your opinion?
4. The aim of the PPA awareness day was to increase awareness that people with PPA benefit from speech and language therapy. Do you think this was clear? (1= not so clear, 10= very clear)
5. How do you think we could spread this message more, differently or better?
6. What would you like next years International PPA awareness day to focus on?
7. Is there anything else you would like to tell us to make future International PPA awareness days even better?

**Appendices 2: Micro costing - Facilitator cost diaries**

| **Activities undertaken by AV and RT:** | **Hours** |
| --- | --- |
| Meetings to plan PPA awareness day related activities | 25 |
| Attending PPA awareness activities e.g., podcasts/webinars | 6 |
| Time spent writing materials, slides, etc | 25 |
| Meetings with RCSLT to plan for Houses of Parliament event  Actual event | 9  5 |
| Meetings with people with PPA – BCPPA PPI advisory group | 6 |
| Meetings with international collaborators | 4 |
| Actual PPA Awareness Day webinar | 2 |
| **Total hours / cost** | **82 hours = *£6,702.844*** |
| Additional other costs: | |
| Travel costs for RT | £60 |
| Costs associated with Houses of Parliament event (Catering, room hire and photographer) | £1,407 |
| Financial reimbursement for BCPPA PPI advisory group including honorarium, travel costs and refreshments | £1500 |
| ***Total:*** | ***£9,669.844*** |

***Sensitivity Analysis for the extra costing for future webinars***

| *Consultant Neurologist: 2 hrs at £103 cost per working hour including qualifications (ref:* <https://kar.kent.ac.uk/109563/> *)* | *£206* |
| --- | --- |
| *Speech and language therapist: 2 hours at £77 cost per working hour including oncosts and overheads (PSSIU cost year 2023/24 – currently most to date rates) (ref:* <https://kar.kent.ac.uk/109563/> *)* | *£154* |
| *Rare Dementia Support: 2 hours* at £26.67 | £53.34 |
| *PPI costs: Four PPI contributors £25 p/hr per person for the 2 hour webinar plus one hour preparation and one hour debrief (ref:* <https://www.nihr.ac.uk/payment-guidance-researchers-and-professionals> *)* | *£400* |
| ***Total:*** | ***£813.34*** |
